# Supplementary figures and images for: Unveiling the Genomic Basis of Chemosensitivity in Sarcomas of the Extremities: An Integrated Approach for an Unmet Clinical Need
Source: Int J Mol Sci. 2023 Apr 8;24(8):6926. doi: 10.3390/ijms24086926 (PMC10138892; doi:10.3390/ijms24086926)

## Slide 1
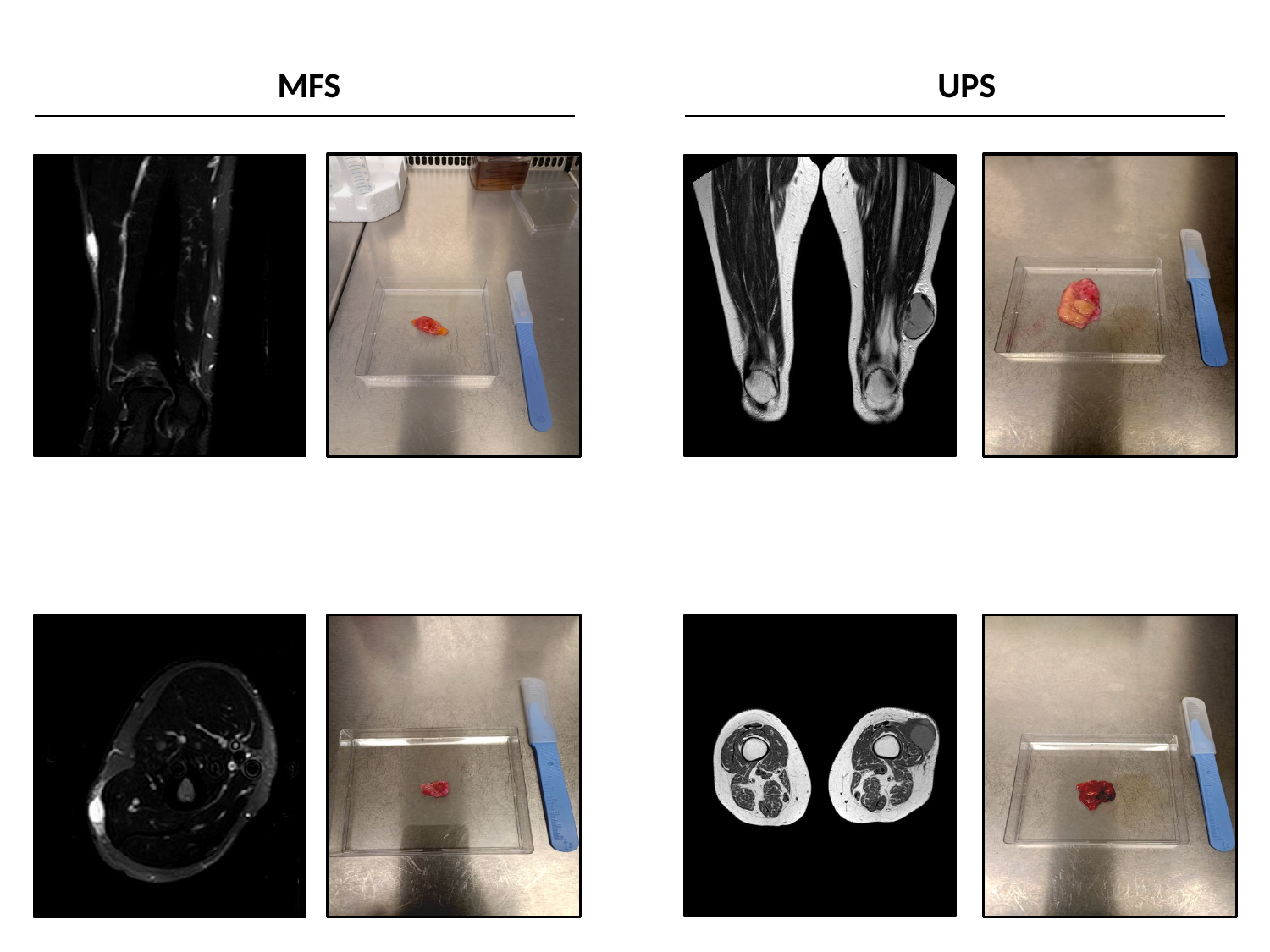

MFS
UPS

Supplement: Supplementary file 1 [file ijms-24-06926-s001.zip › Supplementary Figure S1.pptx]

## Slide 1
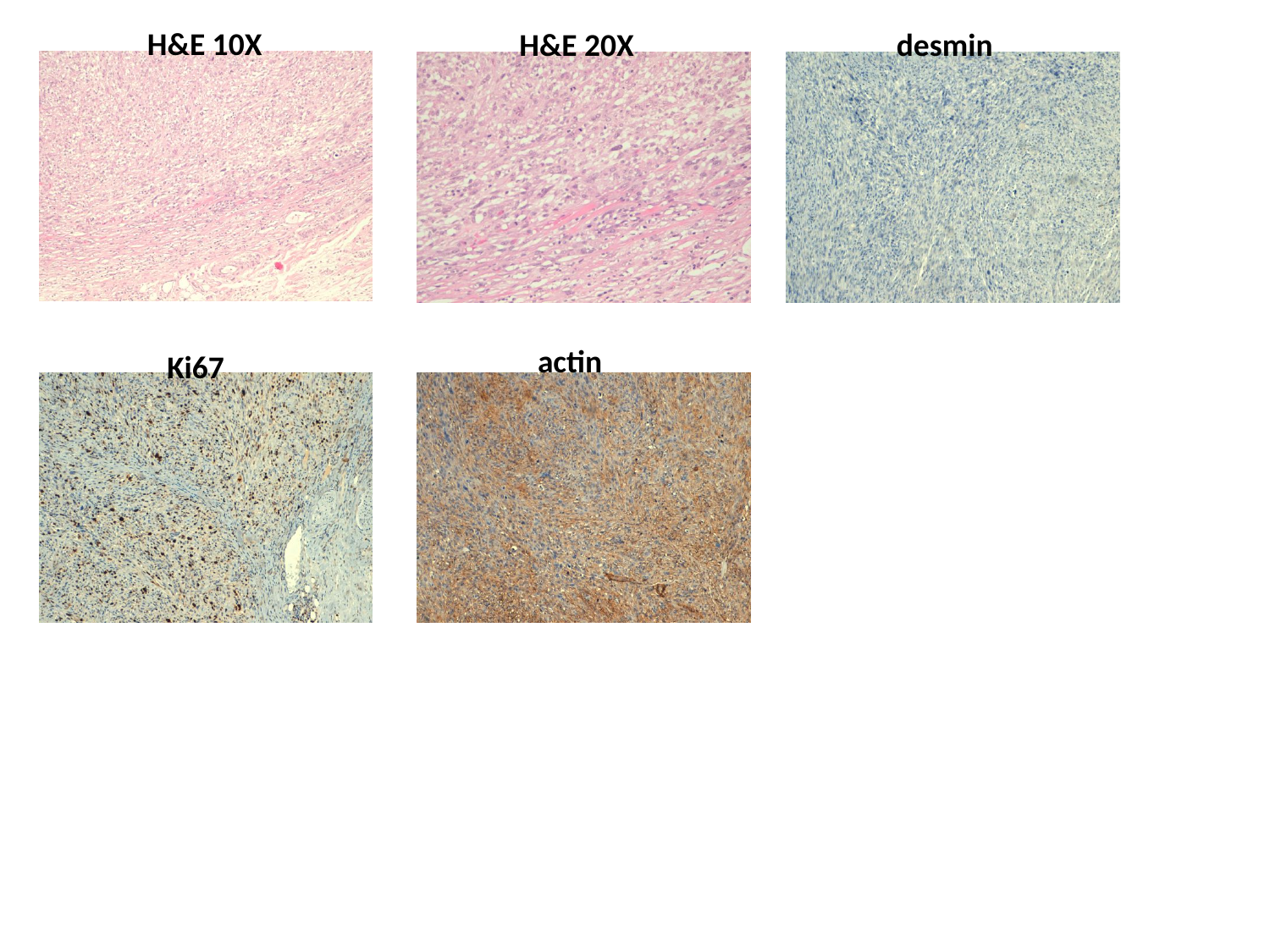

H&E 10X
H&E 20X
desmin
actin
Ki67

Supplement: Supplementary file 1 [file ijms-24-06926-s001.zip › Supplementary Figure S2.pptx]

## Slide 1
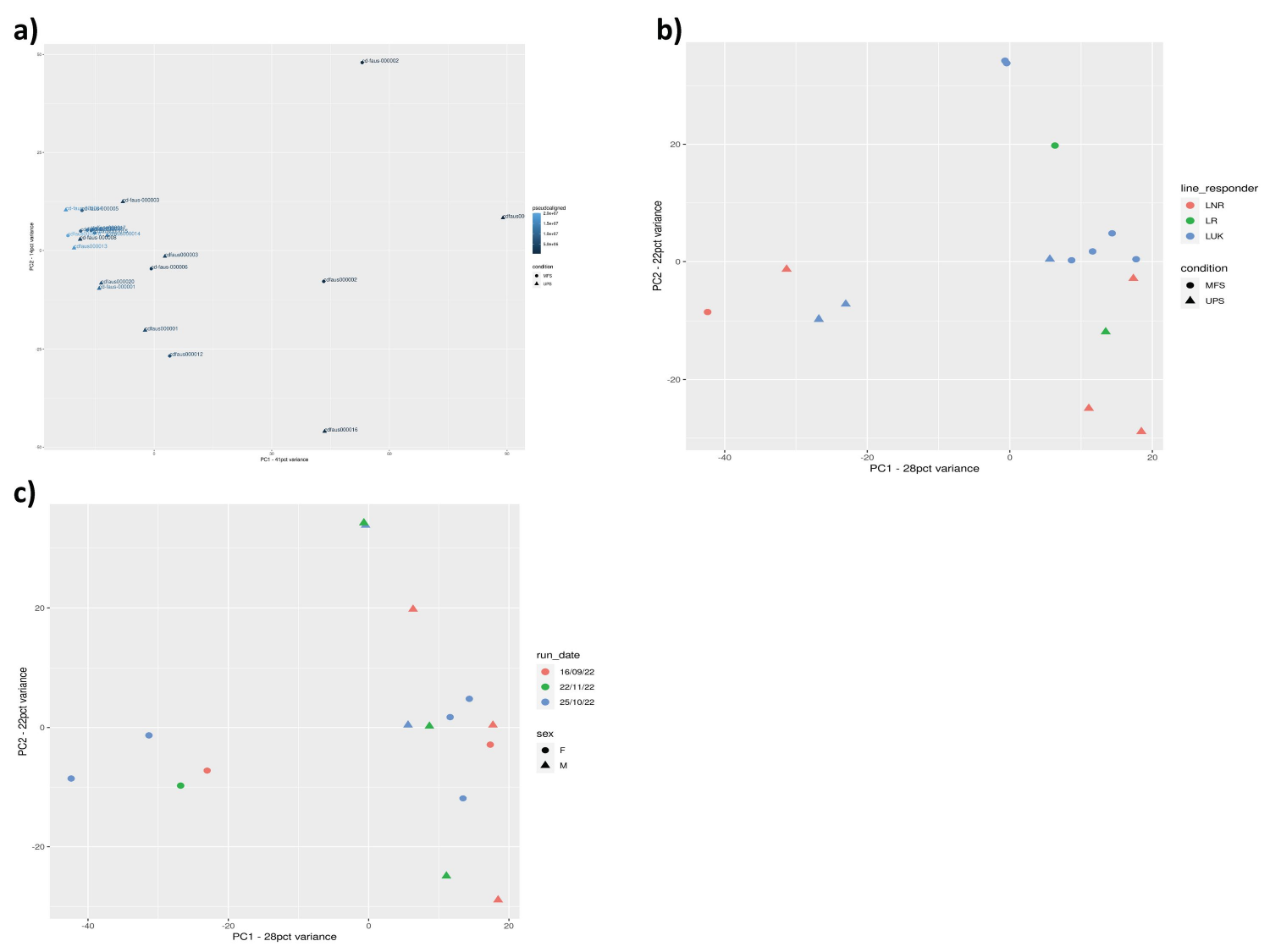

Supplement: Supplementary file 1 [file ijms-24-06926-s001.zip › Supplementary Figure S3.pptx]

## Slide 1
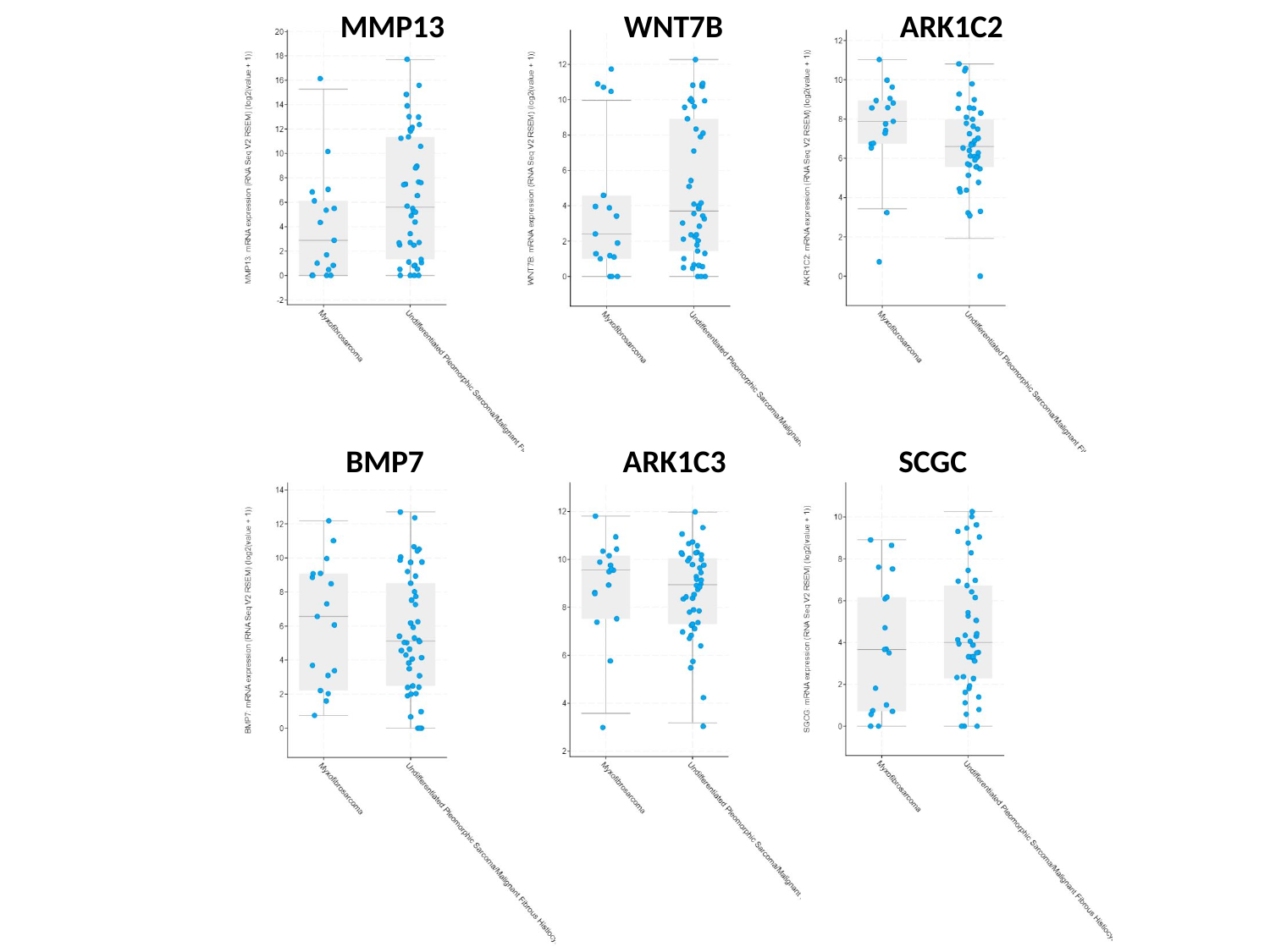

MMP13
WNT7B
ARK1C2
BMP7
ARK1C3
SCGC

Supplement: Supplementary file 1 [file ijms-24-06926-s001.zip › Supplementary Figure S4.pptx]
